# Supplementary material for: Unveiling the Bioactive Potential of the Invasive Jellyfish Phyllorhiza punctata Through Integrative Transcriptomic and Proteomic Analyses
Source: Biomolecules. 2025 Aug 4;15(8):1121. doi: 10.3390/biom15081121 (PMC12383608; doi:10.3390/biom15081121)
Supplement: Supplementary file 1 [file biomolecules-15-01121-s001.zip › Suplementary Material/FileS3/PEPFOLD4/XP_065055791_15858/clusters.html]

 PEP-FOLD clusters


Download archive of all models  here   

|  |  |  |
| --- | --- | --- |
| # | file | sOPEP |
| # cluster 1 - Size: 22 |  |  |
|  | XP\_065055791\_15858-model1 | -12.9535 |
|  | XP\_065055791\_15858-model1.1 | -12.9535 |
|  | XP\_065055791\_15858-model1.2 | -12.9535 |
|  | XP\_065055791\_15858-model1.3 | -12.9535 |
|  | XP\_065055791\_15858-model1.4 | -12.9535 |
|  | XP\_065055791\_15858-model1.5 | -12.9535 |
|  | XP\_065055791\_15858-model1.6 | -12.9535 |
|  | XP\_065055791\_15858-model1.7 | -12.9535 |
|  | XP\_065055791\_15858-model1.8 | -12.9535 |
|  | XP\_065055791\_15858-model1.9 | -12.9535 |
|  | XP\_065055791\_15858-model1.10 | -12.9535 |
|  | XP\_065055791\_15858-model1.11 | -12.9535 |
|  | XP\_065055791\_15858-model1.12 | -12.9535 |
|  | XP\_065055791\_15858-model1.13 | -12.9535 |
|  | XP\_065055791\_15858-model1.14 | -12.9535 |
|  | XP\_065055791\_15858-model1.15 | -12.9535 |
|  | XP\_065055791\_15858-model1.16 | -12.8159 |
|  | XP\_065055791\_15858-model1.17 | -12.8159 |
|  | XP\_065055791\_15858-model1.18 | -12.8159 |
|  | XP\_065055791\_15858-model1.19 | -12.8159 |
|  | XP\_065055791\_15858-model1.20 | -12.8159 |
|  | XP\_065055791\_15858-model1.21 | -12.8159 |
| # cluster 2 - Size: 3 |  |  |
|  | XP\_065055791\_15858-model2 | -12.8945 |
|  | XP\_065055791\_15858-model2.1 | -12.8945 |
|  | XP\_065055791\_15858-model2.2 | -12.8017 |
| # cluster 3 - Size: 6 |  |  |
|  | XP\_065055791\_15858-model3 | -12.8328 |
|  | XP\_065055791\_15858-model3.1 | -12.8328 |
|  | XP\_065055791\_15858-model3.2 | -12.8328 |
|  | XP\_065055791\_15858-model3.3 | -12.8328 |
|  | XP\_065055791\_15858-model3.4 | -12.8328 |
|  | XP\_065055791\_15858-model3.5 | -12.8328 |
| # cluster 4 - Size: 65 |  |  |
|  | XP\_065055791\_15858-model4 | -12.829 |
|  | XP\_065055791\_15858-model4.1 | -12.829 |
|  | XP\_065055791\_15858-model4.2 | -12.829 |
|  | XP\_065055791\_15858-model4.3 | -12.829 |
|  | XP\_065055791\_15858-model4.4 | -12.829 |
|  | XP\_065055791\_15858-model4.5 | -12.829 |
|  | XP\_065055791\_15858-model4.6 | -12.829 |
|  | XP\_065055791\_15858-model4.7 | -12.829 |
|  | XP\_065055791\_15858-model4.8 | -12.829 |
|  | XP\_065055791\_15858-model4.9 | -12.829 |
|  | XP\_065055791\_15858-model4.10 | -12.829 |
|  | XP\_065055791\_15858-model4.11 | -12.829 |
|  | XP\_065055791\_15858-model4.12 | -12.829 |
|  | XP\_065055791\_15858-model4.13 | -12.829 |
|  | XP\_065055791\_15858-model4.14 | -12.829 |
|  | XP\_065055791\_15858-model4.15 | -12.829 |
|  | XP\_065055791\_15858-model4.16 | -12.829 |
|  | XP\_065055791\_15858-model4.17 | -12.829 |
|  | XP\_065055791\_15858-model4.18 | -12.829 |
|  | XP\_065055791\_15858-model4.19 | -12.829 |
|  | XP\_065055791\_15858-model4.20 | -12.829 |
|  | XP\_065055791\_15858-model4.21 | -12.829 |
|  | XP\_065055791\_15858-model4.22 | -12.829 |
|  | XP\_065055791\_15858-model4.23 | -12.829 |
|  | XP\_065055791\_15858-model4.24 | -12.829 |
|  | XP\_065055791\_15858-model4.25 | -12.829 |
|  | XP\_065055791\_15858-model4.26 | -12.829 |
|  | XP\_065055791\_15858-model4.27 | -12.829 |
|  | XP\_065055791\_15858-model4.28 | -12.829 |
|  | XP\_065055791\_15858-model4.29 | -12.829 |
|  | XP\_065055791\_15858-model4.30 | -12.829 |
|  | XP\_065055791\_15858-model4.31 | -12.829 |
|  | XP\_065055791\_15858-model4.32 | -12.829 |
|  | XP\_065055791\_15858-model4.33 | -12.829 |
|  | XP\_065055791\_15858-model4.34 | -12.829 |
|  | XP\_065055791\_15858-model4.35 | -12.829 |
|  | XP\_065055791\_15858-model4.36 | -12.829 |
|  | XP\_065055791\_15858-model4.37 | -12.829 |
|  | XP\_065055791\_15858-model4.38 | -12.829 |
|  | XP\_065055791\_15858-model4.39 | -12.829 |
|  | XP\_065055791\_15858-model4.40 | -12.829 |
|  | XP\_065055791\_15858-model4.41 | -12.829 |
|  | XP\_065055791\_15858-model4.42 | -12.829 |
|  | XP\_065055791\_15858-model4.43 | -12.829 |
|  | XP\_065055791\_15858-model4.44 | -12.829 |
|  | XP\_065055791\_15858-model4.45 | -12.829 |
|  | XP\_065055791\_15858-model4.46 | -12.829 |
|  | XP\_065055791\_15858-model4.47 | -12.829 |
|  | XP\_065055791\_15858-model4.48 | -12.829 |
|  | XP\_065055791\_15858-model4.49 | -12.829 |
|  | XP\_065055791\_15858-model4.50 | -12.829 |
|  | XP\_065055791\_15858-model4.51 | -12.829 |
|  | XP\_065055791\_15858-model4.52 | -12.829 |
|  | XP\_065055791\_15858-model4.53 | -12.829 |
|  | XP\_065055791\_15858-model4.54 | -12.829 |
|  | XP\_065055791\_15858-model4.55 | -12.829 |
|  | XP\_065055791\_15858-model4.56 | -12.829 |
|  | XP\_065055791\_15858-model4.57 | -12.829 |
|  | XP\_065055791\_15858-model4.58 | -12.829 |
|  | XP\_065055791\_15858-model4.59 | -12.829 |
|  | XP\_065055791\_15858-model4.60 | -12.829 |
|  | XP\_065055791\_15858-model4.61 | -12.829 |
|  | XP\_065055791\_15858-model4.62 | -12.829 |
|  | XP\_065055791\_15858-model4.63 | -12.829 |
|  | XP\_065055791\_15858-model4.64 | -12.829 |
| # cluster 5 - Size: 4 |  |  |
|  | XP\_065055791\_15858-model5 | -12.8255 |
|  | XP\_065055791\_15858-model5.1 | -12.8255 |
|  | XP\_065055791\_15858-model5.2 | -12.8255 |
|  | XP\_065055791\_15858-model5.3 | -12.8255 |
|  |  |  |
